# Current Chemotherapy and Potential New Targets in Uterine Leiomyosarcoma

**DOI:** 10.14740/jocmr2419w

**Published:** 2016-01-26

**Authors:** Shabnam Momtahen, John Curtin, Khush Mittal

**Affiliations:** a Department of Pathology and Laboratory Medicine, Weill Cornell Medical College of Cornell University, New York, NY, USA; b Department of Obstetrics and Gynecology, Division of Gynecologic Oncology, Langone Medical Center, New York University School of Medicine, New York, NY, USA; c Department of Pathology, Langone Medical Center, New York University School of Medicine, New York, NY, USA

**Keywords:** Leiomyosarcoma, Uterus, Chemotherapy, Oncogenes

## Abstract

A variety of chemotherapeutic agents have been used for treating recurrent or advanced stage uterine leiomyosarcoma (ULMS). The response rates of these current agents are disappointing, with partial response rates varying from 0% to 33%, and complete response rates varying from 0% to 8%. Recent studies have documented many molecular changes in ULMSs. Prominent amongst these are gains of growth factors C-MYC, Bcl-2, K-ras, and Ki-67, and losses in tumor suppressors p16, p53, Rb1, ING2 and D14S267. Various techniques that have been used to target these molecules are presented. Targeting specific therapies at these underlying molecular changes could potentially yield better response rates with fewer side effects.

## Introduction

Uterine leiomyosarcoma (ULMS) is the most common type of uterine sarcomas [1, 2] and is a highly malignant disease with 5-year survival rates averaging around 40% [1-6]. ULMS is characterized by early hematogenous spread, with local and distant recurrences [1, 2, 7, 8].

Treatment of choice for ULMS is total abdominal hysterectomy and removal of extra-uterine disease. Adjuvant radiation therapy and chemotherapy have been investigated, but no highly effective agents are currently available. More studies are needed for new treatment strategies and novel targeted therapies for ULMS. Recent findings from genetic changes in ULMS raise the possibility of individualized targeted therapy in cases of ULMS.

This review focuses on chemotherapeutic agents in current use, followed by genetic changes and potential targeted therapeutic agents for ULMS.

## Current Therapeutic Agents

Adjuvant chemotherapy is the mainstay of treatment of advanced or recurrent ULMS. There are only limited data available to support their efficacy.

### Gemcitabine and docetaxel

Gemcitabine is an inhibitor of DNA synthesis and repair. Look and co-workers [9] studied the effects of gemcitabine in 48 patients with ULMS, from which 42 were eligible for evaluation of the efficacy and toxicity of the treatment. Nine patients showed overall objective response. Of these, eight showed partial response, and one showed complete response.

Gemcitabine-docetaxel (a microtubule dynamic suppressor) combination has been evaluated as a second-line therapy in 51 ULMS patients, from which 48 were evaluable for response to therapy [10]. Thirteen patients showed objective overall response. Of these, 10 showed partial response and three showed complete response. Two patients remained progression free after 24 months of follow-up.

In another trial of gemcitabine-docetaxel in 42 patients with metastatic ULMS, 15 of the 39 evaluable patients showed response to therapy [11]. Thirteen showed partial response and two patients showed complete response.

Gemcitabine-docetaxel was evaluated in another cohort of 25 patients, from which 23 were evaluable [12]. Forty percent of these patients (10 out of 25) remained progression free at 2 years, with a median progression-free survival of 13 months.

Overall, gemcitabine-docetaxel has shown complete response in only 5% of patients, with partial response in approximately 20-30% of cases.

### Mitomycin, doxorubicin, and cisplatin (MAP)

Edmonson and co-workers [13] evaluated effectiveness of triple combination chemotherapy of MAP in ULMSs. Mitomycin is an anti-neoplastic antibiotic with alkylating effects while cisplatin is a platinum anti-tumor agent. Of the 41 patients, 35 were evaluable for tumor response. This combination therapy resulted in objective overall response in eight patients, of whom five indicated partial response and the other three showed complete response.

### Topotecan

Topotecan is a topoisomerase-I inhibitor which is evaluated as one of the potential chemotherapeutic agents for ULMS. Assessment of the treatment response rate in 36 patients indicated that four patients revealed objective overall response, with three showing partial response and one showing complete response [14].

### Adriamycin (doxorubicin)

Adriamycin is an anthracycline antibiotic intercalating DNA. Omura et al evaluated adriamycin with and without dimethyl-triazeno-imidazole-carboxamide (DTIC), an alkylating agent that deranges purine synthesis, in the treatment of advanced and recurrent sarcomas of the uterus [15]. Of 28 patients with ULMS who received adriamycin alone, seven had overall objective response (25%), while in 20 patients who received combination therapy of adriamycin and DTIC, six showed overall objective response (30%).

Combination of adriamycin and bevacizumab (a recombinant human monoclonal antibody against VEGF) was assessed in the treatment of 17 patients with soft tissue sarcoma, of whom seven had ULMS [16]. Two out of seven patients showed an overall objective response which was partial in both of them.

In a report of two cases with ULMS treated with combination of doxorubicin, ifosfamide (an alkylating agent), and dacarbazine (an inhibitor of DNA-RNA synthesis), both cases showed overall objective response, one showed partial response and the other had complete response to this combination therapy [17].

Combination therapy of adriamycin and ifosfamide was evaluated in a trial of 33 patients with ULMS [18]. Eleven out of 33 showed objective overall response (nine partial and two complete response).

### Thalidomide

Thalidomide is an anti-angiogenic agent. McMeekin and co-workers found no objective overall response in 30 patients with persistent or recurrent ULMS [19].

### Sunitinib malate

Multi-targeted agents have the advantage of potentially targeting a number of different molecules or pathways. Sunitinib malate is a multi-target small molecule inhibitor of tyrosine kinase. Of the 23 patients evaluable for response to treatment, two showed objective overall response that was partial in both of them [20].

### Trabectedin

Trabectedin (ET-743) is a marine-derived anti-tumor agent which interferes with cell division, genetic transcription, and DNA repair via binding to the minor groove of DNA. In a clinical study of 52 patients with ULMS, partial response was observed in 11 patients [21]. None showed complete response.

Amant et al [22] found ET-743 to be superior to other agents, with median survival of 6 months in persistent or advanced disease. In a case report presentation of a 46-year-old white female who underwent total hysterectomy and left salpingo-oophorectomy for an incidental ULMS, treatment with trabectedin resulted in 14 months of stability with a good performance status and partial response to treatment, followed by progression of thoracic disease after that [23].

### Summary of current therapeutic agents in ULMS

The response rates of currently available agents for ULMS are summarized in Table 1 [9-16, 18-22]. The response rates of these current agents are disappointing, with partial response rate varying from 0% to 33%, and complete response rate varying from 0% to 8%.

**Table 1:** Therapeutic Agents, Objective Response, Partial Response, and Complete Response Rates

Therapeutic agent	Patients, N	OR rate, N, %	PR rate, N, %	CR rate, N, %	Reference
Doxorubicin	41(28 evaluable)	7/28, 25%	NA	NA	Omura et al [15]
Bevacizumab + doxorubicin	7	2/7, 28%	2/7, 28%	0/7, 0%	D’Adamo et al [16]
Doxorubicin + dimethyl-triazeno-imidazole-carboxamide (DTIC)	31(20 evaluable)	6/20, 30%	NA	NA	Omura et al [15]
Trabectedin	3	1/3, 33%	1/3, 33%	0/5, 0%	Amant et al [22]
Trabectedin	52	NA	11/52, 21%	NA	Grosso et al [21]
Gemcitabine	42	9/42, 21%	8/42, 19%	1/42, 2%	Look et al [9]
Gemcitabine + docetaxel	23	Not reached after 49 months	NA	NA	Hensley et al [10]
Gemcitabine +docetaxel (first line)	42 (39 evaluable)	15/39, 38%	13/39, 33%	2/39, 5%	Hensley et al [11]
Gemcitabine + docetaxel (second line)	48	13/48, 27%	10/48, 20%	3/48, 6%	Hensley et al [12]
Ifosfamide + doxorubicin	33	10/33, 30%	9/33, 27%	1/33, 3%	Sutton et al [18]
Mitomycin, doxorubicin, and cisplatin	35	8/35, 22%	5/35, 14%	3/35, 8%	Edmonson et al [13]
Sunitinib	23	2/23, 8%	2/23, 8%	0/23, 0%	Hensley et al [20]
Thalidomide	29	0/29, 0%	0/29, 0%	0/29, 0%	McMeekin et al [19]
Topotecan	36	4/36, 11%	3/36, 8.4%	1/36, 2.7%	Miller et al [14]

OR rate: objective response rate; PR rate: partial response rate; CR rate: complete response rate.

## Genetic and Immunohistochemical Changes in ULMS and Potential Targets for Therapy

Multiple studies have investigated genetic and immunohistochemical changes in ULMS. These studies show that at a genetic level, ULMS is a heterogeneous group, with varied genetic changes in different tumors from different patients. None of the current treatments have used an individualized approach that would target specific genetic changes in leiomyosarcomas. In the next section, we will review the current literature on genetic changes in ULMS and implications of those findings.

### C-MYC

C-MYC gene, located at 8q21.2-q24.23, encodes for a transcription factor, and is amplified in up to 47% of ULMS [24, 25].

MYC proto-oncoprotein has been considered as a potential specific target for cancer therapy [26]. MYC proteins form heterodimers with MAX protein and activate the transcription of different target genes. Deregulated MYC gene disturbs the balance, and leads to abnormal expression of the MYC-target genes. Different types of endogenous MYC/MAX complex antagonists, such as MAX/MAX, MAD/-MAX and Mnt1/MAX, have been found to downregulate transcription of MYC genes, and could serve as potential anti-tumor agents.

Antisense-mediated inhibition of C-MYC has been evaluated. AVI-4126 is a PMO (phosphorodiamidate morpholino oligomer) targeted against C-MYC, and has been extensively studied in animal models and human clinical trials. In a phase I clinical trial, patients undergoing resection surgery for adenocarcinoma of the prostate or breast tumors were given a single dose of 90 mg AVI-4126 PMO. Results of the study indicated significant concentrations of PMO in both prostate and breast tumor tissues [27]. In a separate study, Iversen et al have evaluated the bioavailability and efficacy of AVI-4126 in human prostate cancer xenograft murine model and its safety in a phase I human clinical study. Data revealed 75-80% reduction in tumor burden of animals, and no toxicity or serious adverse events in the phase I of human safety trial [28].

To test the anti-tumor effects of a novel antisense oligodeoxynucleotide (ODN) liposomal formulation, the coated cationic liposomes (CCLs), through the use of a monoclonal antibody against the disialoganglioside GD2, Pastorino et al performed an *in vitro* and *in vivo* study by targeting C-MYC in melanoma. They found that the mice bearing subcutaneous human melanoma xenografts and treated with a GD2-CCL-myc-as, exhibited significantly reduced tumor growth and increased survival [29].

Above findings establish the feasibility of using these specific novel treatments in those ULMSs that show C-MYC overexpression.

### K-ras

K-ras protein is a GTPase that plays an important role in many signal-transduction pathways. Amplification has been reported in 33% (two of six) of ULMSs [29]. An assessment of 12p12.1 however revealed no mutation in this location of human genome in 23 ULMS patients [30].

Several of the therapeutic agents directed against K-ras such as farnesyltransferase inhibitors (FTIs), Raf kinase inhibitors, MEK inhibitors, and mammalian target of rapamycin (mTOR) inhibitors have demonstrated clinical activity.

In a phase I study of CCI-779 (one of the rapamycin-analogue mTOR inhibitors), CCI-779 was well tolerated and effectively inhibited mTOR at doses below the level of toxicity. Evaluation of 111 patients with advanced, refractory renal cell carcinoma in a randomized phase II trial confirmed anti-tumor activity of this agent with an overall response rate of 33%. In phase I study of CI-1040 (one of the MEK inhibitors) in patient with pancreatic cancer, Cl-10140 was well tolerated and resulted in partial response in one patient, and stable disease in 19 of 66 patients (28%). For more investigation on CI-1040, a phase II study has been completed in patients with advanced non-small-cell lung cancer (NSCLC), breast cancer, colon cancer and pancreatic cancer. However, only eight out of 67 treated patients had stable disease (12%) with no complete or partial response. Although several therapeutic agents of K-ras targeted therapies have demonstrated clinical activity, multiple targets have been involved in their anti-tumor effects and it may not be specifically due to K-ras inhibition [31].

Targeting mutated K-ras in 24 patients with pancreatic adenocarcinoma using an adjuvant vaccine revealed that vaccination was safe and tolerable [32].

### Ki-67

MIB-1 is a highly specific monoclonal antibody to Ki-67, which is a nuclear protein and a cellular proliferation marker [33]. We and others have found elevated MIB-1 expression in vast majority of ULMSs [34, 35].

Using nanotechnology mediated subcellular delivery of anti-Ki-67 antibody (TuBB-9) to ovarian cancer cells, inactivation of the proliferation marker pKi-67, and cellular death of proliferating cancerous cells have been achieved [36]. It has been demonstrated that the methylated oligonucleotide targeting Ki-67 promoter has a remarkable effect on the inhibition of Ki-67 expression and the proliferation of the human 786-0 renal carcinoma cells which can induce apoptosis of these cells. These results indicate that using methylated oligonucleotide targeting Ki-67 gene might be a possible approach in the treatment of very high MIB-1 expressing tumors such as leiomyosarcoma [37].

### P53

Alteration in p53 has been reported in about 30-35% of ULMS patients [24, 30, 34, 38-40].

In an analysis of 20 patients with ULMS, six (30%) showed loss of heterozygosity (LOH) in TP53 (17p13.1) [40]. It was previously demonstrated that a modified vaccinia Ankara (MVA) vaccine expressing human p53 (MVA-p53) was moderately active when given as a homologous prime/boost in a human p53 knock in mouse model. Results suggested that p53 protein is an attractive target for an adaptive immune response, in which heterologous p53 immunization induced protection against growth of tumor cells [41].

Re-activation of p53 in tumors that retain a functional p53 is another potential approach in cancer drug therapy. By targeting MDM2, which is a negative regulator of p53, it may be possible to restore p53 function to control tumor growth. MDM2 is a p53 E3 ubiquitin ligase that mediates degradation of p53 while p14/ARF which is an MDM2-binding protein prevents p53 degradation and controls the activity of MDM2. MDMX antagonizes p53-dependent transcriptional control by interfering with p53 transactivation function. Novel approaches that have been tested in a preclinical setting or are currently in clinical development include adenovirus-based p53 gene therapy, small molecules such as PRIMA that can restore the transcriptional transactivation function to mutant p53, and NUTLIN and RITA that interfere with MDM2-directed p53 degradation [42, 43].

### Bcl-2

Bcl-2 (18q21.33) encodes a protein that is an inhibitor of apoptosis in the cell growth cycle, prevents the normal cell death, and leads to uncontrolled cell growth and tumor development. To evaluate the expression of Bcl-2 in ULMS, 21 paraffin-embedded tissue slides were evaluated by immunohistochemistry. Bcl-2 was expressed in 12 of 21 (57%) LMS tissue slides [44].

Bcl-2, its anti-apoptotic relatives MCL-1 and BCL-XL, and the pro-apoptotic BH3-only ligand BIM were found to be coexpressed at relatively high levels in heterogeneous breast tumors. To explore the role of Bcl-2 as a potential therapeutic target in breast cancer, Oakes et al generated a panel of primary breast tumor xenografts in immunocompromised mice and treated them with either ABT-737 (BH3 mimetic), docetaxel, or a combination. They indicated that treatment with ABT-737 alone was ineffective; however, tumor response and overall survival were significantly improved by combination therapy, suggesting that ABT-737 sensitizes the tumor cells to docetaxel [45].

Bcl-2 family of proteins plays an important role in regulating apoptosis in the mammalian cells via its interacting pro- and anti-apoptotic members (Bax and Bcl-2). Inhibition of Bcl-2 proteins is one of the most promising new approaches to targeted therapy in cancer. To study the induction of apoptosis in human fibrosarcoma cells (HT1080) after treatment with a series of ferrocene compounds (FTFs), the investigators found that FTFs could suppress the viability of HT1080 cells through decreasing mitochondrial membrane potential. They indicated that Bax/Bcl-2 ratio in HT1080 cells was significantly increased under the stress of FTFs suggesting that FTFs-induced apoptosis in HT1080 cells may work dependent on a Bax/Bcl-2 pathway [46].

Studies revealed that there is a potential binding site of microRNA-7 (miR-7) on 3’UTR of BCL-2 and it was further shown that Bcl-2 was downregulated by miR-7 at both transcriptional and translational levels. Since miR-7 has been indicated to be a potential tumor suppressor in various cancers, Xiong et al designed a study to evaluate the effects of miR-7 in NSCLC cell lines. In this study, they observed that overexpression of miR-7 suppressed cellular proliferation, induced cell apoptosis and inhibited *in vitro* cell migration, and also reduced *in vivo* tumorigenicity. Their investigation showed that Bcl-2 was downregulated by miR-7 and suggests that Bcl-2 could be a novel target involved in the pathway of miR-7-mediated growth suppression and apoptosis [47]. Oblimersen is one of the Bcl-2 antisense drugs. It reduces Bcl-2 protein translation by degradation of Bcl-2 mRNA. It has been evaluated in a number of clinical trials for different malignancies [48]. Investigation on pancreatic cancer showed that some Bcl-XL (an anti-apoptotic factor of the Bcl-2 family) sequence-specific antisense oligonucleotides could suppress pancreatic tumor growth and sensitize tumor cells to gemcitabine [49].

Small molecule inhibitors of Bcl-2 family such as gossypol (BL193), TW-37, apogossypolone (ApoG2), AT-101, ABT-737, obatoclax (GX-015-070), and HA14-1, have also shown promise in Bcl-2 targeting therapy. These agents bind to the Bcl-2 hydrophobic groove and are thought to inhibit the anti-apoptotic function of Bcl-2 and may be as effective as standard chemotherapeutics agents [50].

### Platelet-derived growth factor (PDGF)

PDGF is an important regulator of cell growth and carcinogenesis. Elimination of tyrosine kinases PDGF receptor results in cell cycle arrest and apoptosis. Anderson et al evaluated genetic changes in 25 patients with ULMS and they indicated that 15 out of 25 (60%) overexpressed PDGF receptor [39]. To evaluate the genetic aberrations in ULMS, we previously examined six cases of ULMS using high-density oligonucleotide array-CGH. Loss of chromosome in 22q13.1-22q13.3 sequence, an area near the gene encoding PDGF was found in four cases (66%) [24]. It was suggested that there are sequences near PDGF gene coding area whose loss may lead to ULMS. Possible role of tyrosine kinase inhibitors has been considered to target PDGF receptor as a potential target for treatment. It has been shown that PDGF receptor activation sensitizes some type of non-gastrointestinal stromal tumor (GIST) sarcoma cells to PDGF receptor kinase inhibitors such as sunitinib [51]. Multiple tyrosine kinase inhibitors like sorafenib, pazopanib, gefitinib, and semaxanib are also under investigation as potential therapeutic agents in different types of human sarcomas.

### P16

P16 is a cyclin-dependent kinase inhibitor which specifically binds to the cyclin-dependent kinase CDK-4 and inhibits the catalytic activity of the CDK4-cyclin D complex, and thereby acts as a negative cell cycle regulator. Although, deletion, mutation or promoter methylation may lead to molecular pathogenesis of diverse types of tumors, the role of p16 in ULMS is not clearly known. Zhai and co-workers assessed LOH at some areas within or close to tumor suppressor genes and they reported p16 (9p21) LOH in six out of 20 (30%) of LMS patients [40]. Similarly, we reported two out of six cases of ULMS (33%) with LOH in 9p21.3 locus [24]. Molecular investigation of the chromosome 9 has also led to the identification of homozygous genetic loss of 9p21.1 and 9p21.3 in another study of this chromosome [52].

To develop novel cancer therapeutics targeting the G1/S control, through inhibition of cyclin-CDK activities, Lukas et al performed a study on P16ink4a and pRb, two components of G1/S regulatory pathway. To evaluate the effects of wild-type p16 and pRb on cell cycle progression over an extended period of time, they performed transient gene transfer experiments with human U-2-OS (human osteogenic sarcoma cell line) and R12 cells (derived from Rat-1 diploid fibrobroblasts) followed for 60 ± 72 h. It was indicated that G1 arrest which was imposed by wild-type p16 and pRb at 24 h after transfection, remained unchanged for the duration of the entire 72 h experiment only in p16 transfected cells [53].

On the other hand, overexpression of p16 has also been reported as a possible mechanism of tumor development. To compare p16 overexpression in ULMSs and leiomyomas or smooth muscle tumors of uncertain malignant potential, O’Neill et al assessed the p16 immunohistochemical expression in a variety of uterine smooth muscle tumors. They have reported that 19 out of 22 (86%) ULMS overexpressed p16. This was significantly higher than leiomyomas or smooth muscle tumors of uncertain malignant potential [34]. They suggested that the role of p16 in the pathogenesis of malignant uterine smooth muscle tumors may be due to a non-HPV-related mechanism. In another similar study done by Bodner-Adler and co-workers, p16 expression was seen in 12 of 21 (57%) ULMSs [54].

### C-Kit

The c-Kit gene is located on the long arm of chromosome 4 (4q11-12). It encodes a 145-kDa tyrosine kinase receptor in the cell membrane, which is involved in transduction of cell signals. C-Kit expression has been evaluated in multiple studies. Given the therapeutic activity of imatinib (STI571) in GISTs, Raspollini et al have been encouraged to evaluate the expression of c-Kit gene and any possible correlation in 32 patients with ULMS. Overexpression of c-Kit was found without mutation in 17 of 32 evaluated cases (53%). Based on these findings, they hypothesized a potential role for STI571, which is a kit tyrosine kinase inhibitor in the treatment of ULMS [55]. C-Kit overexpression was also assessed in other studies. Wang et al examined c-Kit expression in uterine endometrial stromal sarcomas, leiomyomas, and leiomyosarcomas. Among 16 patients with ULMS, 12 (75%) showed c-Kit overexpression [1]. In another similar study, c-Kit overexpression was seen in 15 of 22 (68%) patient with ULMS [56]. Evaluation of c-Kit overexpression in a case series of 11 patients with different uterine sarcomas indicated that all were positive for c-Kit overexpression [57].

In a study on a series of 18 ULMS cases, DNA extraction and sequencing for exons 11 and 9 of c-Kit was surprisingly negative for any mutations. Since Kit needs to be phosphorylated to start its signaling cascade, and that the ULMSs included in this study showed no phosphorylation of Kit, they suspected the role of c-Kit and anti-tyrosine kinase drug therapy in the ULMS [58]. This suspicion led to a search for presence of any mutation in exon 11 and exon 17 in ULMS. All seven tumors expressed Kit protein at varying levels as assessed by immunohistochemistry. No mutation in exons 11 or 17 of c-Kit was identified in six of seven tumors. Only one of them had deletion of both exons 11 and 17. The authors suggested that in spite of c-Kit overexpression, lack of activating mutation in exon 11 or 17 of c-Kit may diminish any response to imatinib mesylate therapy [59].

As an oral multitargeted tyrosine kinase inhibitor, sunitinib malate has antiangiogenic and anti-tumor activity, which potently inhibits c-Kit receptor kinase and is recommended for treating imatinib-refractory GIST. GISTs are similar to ULMS in expression of c-Kit and resistance to chemotherapeutic treatments. Although the potential role of the treatment with Kit tyrosine kinase inhibitors is currently unknown, different trials have investigated this possibility.

Successful STI571 therapy in patients with advanced GISTs encourages the hope that other sarcomas can be treated in a similar way [60, 61]. However, the assessment of the efficacy and toxicity of sunitinib in women with recurrent and persistent ULMS has not indicated hopeful results [20].

### Chromosome 9 and 11

Loss of genetic contents in both 9p22.1 and 9q22.3 has been reported [40, 52]. In addition, 9q33.1-34.2 sequence, which encodes endoglin, a tumor suppressor gene, showed loss in 33% of evaluated ULMS cases [24].

Regarding chromosome 11, two out of six (33%) cases revealed genetic loss in the sequence of 11p14.3-p15 [24], while 11p13 (WT1), which codes for a tumor suppressor gene, was lost in two of 20 cases (10%) [40].

### Chromosome 1, 2, 4, 7, 13, 14, 16, and 18

Zhai et al assessed LOH in different loci within or close to tumor suppressor genes. Locus 18q21.3 (DCC) showed LOH in three out of 20 cases (15%) of LMS. However, evaluation of 18q21.1 (DPC4) showed no genetic loss in this tumor suppressor gene sequence [40].

Zhai et al reported LOH at 13q14.2 (RB1) in six out of 20 (30%) similar to Mittal et al indicating two out of six cases with LOH (33%) [24, 40].

14q32.1 (D14S267) is another tumor suppressor gene sequence which is found to have LOH in eight of 29 cases of ULMS in Zhai et al’s study [40]. Cho et al reported that there is high level of homozygous loss at 14q32.33 [52] and Mittal et al indicated LOH in three out of six (50%) of ULMS cases at 14q13.12-q13.43 [24].

Chromosomal gain was seen in 33% of ULMS cases at 1p21.3-31.3 and may play an important role as an oncogene through early response transcription pathway [24]. Loss of chromosomal homozygosity has been also reported in 57% of ULMS cases at 1p21.1 [52]. Gain has been reported in 1q22-q24.3 (Csk1) in 33% of ULMS cases. Csk1 is a proposed breast cancer oncogene which inhibits apoptosis [24].

Gain in 7q33-35 and 7q36.3 was seen in 57% and 40% of seven ULMS cases, respectively [52].

ING2 (4q34.3-q35.2) is a tumor suppressor gene that has been reported to show loss in 50% of evaluated ULMS cases [24]. Loss in 2q36.1-q37.2 was seen in 33% of ULMS cases [24].

### MDM2

MDM2 (12q13-14), which is a p53 associated protein, was found to be overexpressed in three of 23 (13%) ULMSs [30]. 12q13-15 genetic gain was found in another study as well [52]. ELK3 (12q21.2-q24.3), which is an important factor in activation of transcription in the presence of Ras proto-oncogene, revealed gain of the genetic content [24]. The same finding was reported in a study by Cho et al in 12q23.3 sequence [52].

### Epidermal growth factor receptor (EGFR)

Evaluation of another regulator of cell cycle growth, EGFR, revealed that only one out of 25 ULMS cases (4%) had EGFR expression [39].

### Fumarate hydratase

Alterations were seen in only one out of 67 cases of ULMS in one study [62] and in none of the 22 cases in another study [56].

### Summary of potential new targets in ULMS

A number of potential molecular targets have been identified in ULMS. Prominent amongst these are gains of growth factors C-MYC, Bcl-2, K-ras, and Ki-67, and losses in tumor suppressors p16, p53, Rb1, ING2 and D14S267. These gains and losses are variably present in different ULMS cases. A number of preclinical and clinical studies are examining ways to target these molecular changes in other malignancies. If suitable agents are found, these could be applied to ULMS in a targeted manner, hopefully enhancing effectiveness of chemotherapy. Potentially, molecular changes in tumor cells could also be used to better target conventional chemotherapy to these tumor cells. In a recent study, presence of C-MYC amplification was used to target gemcitabine to human colon cancer grafted in mice, enhancing the effectiveness of this drug [63].
